# Self- and Other-Oriented Motives, Prosociality, and Well-Being in Older Adults: An ESM Study

**DOI:** 10.1093/geroni/igaf122.1879

**Published:** 2025-12-31

**Authors:** Jeanne Nakamura, Ajit Mann

**Affiliations:** Claremont Graduate University, Claremont, California, United States; Loyola Marymount University, Los Angeles, California, United States

## Abstract

In past research on other-related and self-related motives for volunteering, other-related motives have been associated with higher wellbeing. However, some theories of motivation would suggest that self-oriented motives, notably immediate experiential rewards, will co-occur with other-oriented motivation for older adults engaging in prosocial activity. We report research that contributes to the understanding of motivation, prosociality, and well-being in three ways. We examined the conjunction of self- and other-oriented motives in addition to their separate occurrence, and compared how these motives were linked to well-being. Because prosocial behavior, motivation, and well-being fluctuate intra-individually, we examined their relationship using the experience sampling method. Finally, given challenges with researcher classification of volunteering motives as self- or other-oriented, we studied participant-reported motives. Data came from a sample of older, high-commitment volunteers and leaders in social-purpose programs in the US (n of responses=5,501; N = 165, 58% female, age: M = 71.1, SD = 5.7). Consistent with past research, in moments when only other-oriented motivation was reported, positive correlates including eudaimonic well-being and positive affect were higher than when only self-oriented motivation was reported. Notably, a similar pattern held on occasions when other-oriented motivation co-occurred with self-oriented motivation. Theoretical and applied implications of the findings are discussed.

